# Angiotensin Receptors Heterodimerization and Trafficking: How Much Do They Influence Their Biological Function?

**DOI:** 10.3389/fphar.2020.01179

**Published:** 2020-08-03

**Authors:** Natalia L. Rukavina Mikusic, Mauro G. Silva, Angélica M. Pineda, Mariela M. Gironacci

**Affiliations:** Universidad de Buenos Aires, Facultad de Farmacia y Bioquímica, Dpto. Química Biológica, IQUIFIB (UBA-CONICET), Buenos Aires, Argentina

**Keywords:** angiotensin type 1 receptor, angiotensin type 2 receptor, Mas receptor, G-protein–coupled receptor, trafficking, heteromerization, nucleus

## Abstract

G-protein–coupled receptors (GPCRs) are targets for around one third of currently approved and clinical prescribed drugs and represent the largest and most structurally diverse family of transmembrane signaling proteins, with almost 1000 members identified in the human genome. Upon agonist stimulation, GPCRs are internalized and trafficked inside the cell: they may be targeted to different organelles, recycled back to the plasma membrane or be degraded. Once inside the cell, the receptors may initiate other signaling pathways leading to different biological responses. GPCRs’ biological function may also be influenced by interaction with other receptors. Thus, the ultimate cellular response may depend not only on the activation of the receptor from the cell membrane, but also from receptor trafficking and/or the interaction with other receptors. This review is focused on angiotensin receptors and how their biological function is influenced by trafficking and interaction with others receptors.

## Introduction

The renin-angiotensin system (RAS) exerts a fundamental role in blood pressure control and fluid homeostasis. Cardiovascular diseases have been associated with disorders in the RAS. The RAS is composed of two arms. The pressor arm, constituted by angiotensin (Ang) II and the Ang II type 1 receptor (AT1R), which mediates the pressor, trophic, proinflammatory, fibrotic, oxidative effects, among others, of Ang II. The opposite arm, the depressor arm of the RAS, is constituted by Ang-(1-7), its receptor Mas (MasR), which mediates the depressor, vasodilatory, anti-inflammatory, and antifibrotic effects of Ang-(1-7), and the Ang II type 2 receptor (AT2R), which counteracts AT1R-mediated actions (Forrester et al., 2018; Santos et al., 2018). Another component of the RAS, alamandine, should be included in this depressor arm of the RAS. Alamandine induces protective effects as those elicited by Ang-(1-7) but through the stimulation of the Mas-related G-protein–coupled receptor (GPCR) member D receptor (MrgDR) (Lautner et al., 2013; Schleifenbaum, 2019).

MasR, AT1R, AT2R, and MrgDR belong to the GPCRs family. GPCRs comprise the largest and most varied family of transmembrane receptors, with almost 1000 members identified in the human genome (Thomsen et al., 2018). GPCRs are targets for ∼30% of currently approved and clinical prescribed drugs. All GPCRs share a common structure, which consists of one polypeptide that crosses the membrane seven times, with a highly variable extracellular N-terminus and intracellular C-terminus (Sposini and Hanyaloglu, 2018). GPCRs also share the ability to interact with G-proteins, showing a great diversity in the functional coupling and the number of alternative signaling pathways elicited by their activation (Fredriksson et al., 2003).

GPCRs comprise the most important receptor family that participates in signaling in response to extracellular stimuli in eukaryotes (Calebiro and Godbole, 2018). Given their fundamental role in cell physiology, GPCR signaling is a tightly regulated process, influenced by multiple factors in a spatial and temporal way. Ligand availability, the properties of both the receptor and G-protein, the interaction with other proteins or receptors, and the trafficking are among the most recognized factors influencing GPCR signaling (Foster and Brauner-Osborne, 2018; Carbone et al., 2019).

GPCR primary mechanism of signaling is *via* coupling to G-proteins, which are heterotrimeric proteins composed by three subunits: Gα, Gβ, and Gγ (Sposini and Hanyaloglu, 2018). Upon cell surface receptor binding to its extracellular ligand, Gα and Gβγ subunits of G-protein dissociate, acquiring the capacity to initiate a signaling cascade of downstream events. Gα proteins are divided into four subfamilies with differential signaling features. Gβγ subunits are numerous and have both regulatory and signaling properties (Wootten et al., 2018). In this prototypic model of GPCR signaling, GPCR activation converges on common downstream signal pathways (Sposini and Hanyaloglu, 2018).

Besides their canonical mechanism of signaling, studies over the past decades have proven that GPCRs mediate diverse physiological functions by activation of pleiotropic signaling mechanisms. These pleiotropic signaling mechanisms have helped to understand the fundamental role of GPCRs in cellular physiology (Hanyaloglu, 2018). These alternative signaling models exhibit extensive signal crosstalk and diversity, allowing GPCRs to interact with an enormous variation of ligands as photons, odorants, tastings, and many hormones and neurotransmitters mediating most physiological and pathological processes (Thomsen et al., 2018; Wootten et al., 2018).

One of the mechanisms by which GPCRs can diversify their signaling is through the interaction with another receptor to associate as either homomeric or heteromeric complexes. Recently, Ferre et al. (2020) defined receptor heteromer as “a macromolecular complex composed of at least two (functional) receptor units (protomers) with biochemical properties that are demonstrably different from those of its individual components.” This definition is different from that of heteromeric receptor, “a dimeric or oligomeric receptor for which the minimal functional unit is composed of two or more different subunits that are not functional on their own” (Ferre et al., 2020). Receptor homomer and homomeric receptor would be, respectively, defined in the same way as receptor heteromer and heteromeric receptor but with the distinction of being formed by “two or more identical protomers or identical non-functional subunits” (Ferre et al., 2020). Heteromerization of receptors may result in alterations in their biosynthesis, plasma membrane diffusion rate, ligand binding, pharmacology, and signaling (Sleno and Hebert, 2018; Sposini and Hanyaloglu, 2018; Bourque et al., 2020).

On the other hand, over the last years, studies have pointed out a significant role of endocytic trafficking as a mechanism underlying GPCR signaling complexity and specificity. Being highly integrated with the GPCR signaling network, endocytic trafficking provides an important system that influences the organization and direction of receptor signaling (Hanyaloglu, 2018).

Thus, the ultimate cellular response may depend not only on the activation of the receptor from the cell membrane but also from receptor trafficking and/or the interaction with other receptors. This review is focused on angiotensin receptors and how their biological function is influenced by trafficking and interaction with others receptors.

## Brief Review of GPCR Signaling History

Although GPCRs play a fundamental role in eukaryotic cell physiology, a vast number of years of exhaustive work were required to unravel the intricate pathways and individual components involved in the process of GPCRs signaling. Interestingly, components of the membrane effector and transduction system were identified before the GPCRs could be isolated and characterized.

In the late 1950s and early 1960s, Earl Sutherland´s work led to the identification of cyclic adenosine monophosphate (cAMP) and adenylate cyclase (AC), which was initially postulated as the receptor of the signaling system (Kresge et al., 2005). This discovery gave him the Nobel Prize for Physiology or Medicine in 1971. The subsequent work of Martin Rodbell and Alfred G. Gilman led to the identification and purification of another component of the system, the membrane effector, which was named as G-protein (Gilman, 1995; Rodbell, 1995). For this, Rodbell and Gilman were given the Nobel Prize for Physiology or Medicine in 1994. One of the main contributions to the knowledge of GPCRs structure and signaling mechanism was that of Robert J. Lefkowitz and colleagues who purified the β-adrenergic receptor (β-AR) for the first time and postulated a ternary complex model to explain the agonist binding to the receptor (De Lean et al., 1980; Lefkowitz, 2004).

During 1970 and 1980, the discovery and implementation of novel experimental techniques such as radioligand binding assay, detergent solubilization, and purification by affinity chromatography led to the cloning of the genes encoding the β2-AR (Dixon et al., 1986). Two important discoveries emerged from the receptor cloning. First, the gene encoding the β2-AR was intronless, meaning that the receptor’s sequence of amino acids could be inferred from one exon (Kobilka et al., 1987). On the other hand, the β2-AR sequence showed homology with the visual pigment rhodopsin, leading to the postulation that all GPCRs might present the same structural arrangement (Dixon et al., 1986).

In parallel with these findings, Lefkowitz and colleagues proposed a mechanism for β2-AR signaling regulation by protein kinase A (PKA) phosphorylation, which was named as heterologous desensitization (Benovic et al., 1985). Further studies showed that the β2-AR could also be phosphorylated in a PKA-independent manner, leading to the identification of the β-adrenergic receptor kinase (βARK), now recognized as G-Protein-coupled receptor kinase 2 (GRK2) (Benovic et al., 1986). Subsequently, a non-visual arrestin named β-arrestin was identified, with the ability to binding to the complex formed between the phosphorylated β2-AR and βARK in order to block its interaction with the G-protein (Benovic et al., 1987). Soon after that, β-arrestin was cloned for the first time (Lohse et al., 1990). Today, it is widely accepted that β-arrestins and GRKs constitute a universal mechanism shared by GPCRs to regulate their signaling.

In later years, several members of the GPCR family were identified. The use of the techniques of mutagenesis and the creation of receptor chimeras improved the knowledge of GPCRs structure and the understanding of many aspects in the regulation of receptor signaling (Prazeres and Martins, 2015).

## Mechanisms of GPCR Signaling Diversification

### Angiotensin Receptors Heteromerization

GPCRs exist as homo-oligomers, in addition to interact with other receptors, forming hetero-oligomers, affecting in this way their functionality. Due to GPCR oligomerization, different properties such as synthesis, cell membrane diffusion, binding to the agonist, pharmacology, signaling, and trafficking may be altered (Milligan, 2010; Ferre et al., 2014; Gomes et al., 2016; Bourque et al., 2020). Due to GPCR oligomerization ligand affinity for its receptor may change. Alternatively, agonist-mediated receptor activation may be counteracted by the antagonist of the other receptor forming the oligomer or one of the protomer forming the heteromer may directly modulate the other protomer resulting in changes in its properties (Farran, 2017). By mediating several unique different effects, GPCR heteromerization plays a fundamental role in cell physiology. GPCR heteromerization has also been implicated in the pathophysiology of several diseases, including cardiovascular and neurological diseases (AbdAlla et al., 2009; Fernández-Dueñas et al., 2019).

Regarding the RAS, some of the responses mediated by AT1R activation are due to heteromerization with other receptors. It has been largely reported heteromerization between the AT1R and the bradykinin type 2 receptor (B2R) (AbdAlla et al., 2000; Hansen et al., 2009; Quitterer et al., 2011; Wilson et al., 2013; Quitterer et al., 2019). AT1R-B2R heteromerization is involved in the increase of Ang II hypersensitivity in preeclampsia (AbdAlla et al., 2000). Preeclampsia is a pregnancy complication characterized by high blood pressure and proteinuria of ≥300 mg/day. It may cause serious complications for the mother and fetus, which it even may be fatal (Bokslag et al., 2016; El-Sayed, 2017). Preeclampsia is associated with vasoconstriction and microthrombi formation, leading to maternal organ reduced blood flow and an increase in the risk of multi-organ dysfunction. As a consequence of placenta hypo-perfusion, complications and growth retardation of the fetus may occur (El-Sayed, 2017). The presence of AT1R-B2R heteromers has been reported in human placental biopsies from pregnancies with preeclampsia (Quitterer et al., 2019). Due to AT1R-B2R heteromerization, the arrestin-dependent internalization of B2R in primary vascular smooth muscle cells (VSMCs) is blocked when the AT1R is stimulated with a specific agonist (Wilson et al., 2013). AT2R can also dimerize with B2R resulting in an enhancement of nitric oxide (NO) production in rat pheochromocytoma cells (Abadir et al., 2006).

Heteromerization between AT1R and AT2R has been reported to be present in rat fetal fibroblasts and in myometrial biopsies from humans (AbdAlla et al., 2001). The interaction results in the inhibition of the inositol phosphate generation induced by AT1R activation, leading to a lower AT1R-mediated response (AbdAlla et al., 2001). In transfected HeLa cells, AT2R inhibits the signaling of AT1R that is induced by the ligand through a pathway dependent on protein kinase C (PKC) activation, and this effect results from constitutive AT1R-AT2R heteromerization (Inuzuka et al., 2016). In proximal tubule cells from pig kidney (LLC-PK1 cells), it has been shown that Ang II internalized together with AT1R-AT2R heteromers forming a complex in a process that was dependent on microtubules but not on clathrin to target endoplasmic reticulum, where it might increase sarco(endo)plasmic reticulum calcium ATPase activity and calcium levels (Ferrao et al., 2017). AT1R endocytosis is also influenced by interaction with other receptors. AT1R internalization is modified due to AT1R-B2R heteromerization. The rate of AT1R-B2R endocytosis is increased compared to B2R alone but slowed compared to AT1R alone (Wilson et al., 2013; Bian et al., 2018). In addition, AT1R-B2R heteromer stimulated by an AT1R agonist leads to a reduction in bradykinin responsiveness (Wilson et al., 2013).

Not only AT2R may antagonize AT1R-mediated actions. The Ang-(1-7) MasR can also antagonize AT1R functional activity in transfected CHO-K1 cells through the formation of a constitutive heterodimer that was unaffected by the presence of their ligands (Kostenis et al., 2005). In fact, Ang II–mediated vasoconstriction is enhanced in vessels from MasR-knockout (KO) mice (Kostenis et al., 2005). Ang II binding to AT1R is diminished due to oligomerization with apelin receptor in transfected human embryonic kidney 293 (HEK293) cells (Siddiquee et al., 2013), whereas ligand occupation of prostaglandin F2α receptor increased Ang II affinity for AT1R in VSMC (Goupil et al., 2015). Heteromerization between the cannabinoid type 1 receptor and AT1R results in an enhanced calcium and mitogenic response to Ang II in hepatic stellate cells isolated from rats (Rozenfeld et al., 2011). Conversely, calcium response to Ang II was attenuated by heteromerization between AT1R and the dopamine type 2 receptor (D2R) in rat striatum and in transfected HEK293T cells (Martinez-Pinilla et al., 2015). Moreover, AT1R blockade by the AT1R antagonist candesartan prevented D2R-mediated effects on cAMP levels, activation of mitogen-activated protein kinase (MAPK), and β-arrestin recruitment. The authors suggested that this crosstalk could have a beneficial effect to prevent the side effects in patients with abnormal motor control and dyskinesia subjected to dopamine-replacement therapy (Martinez-Pinilla et al., 2015).

A crosstalk between the sympathetic nervous system and the RAS has been described (Barki-Harrington et al., 2003), which may be explained at least in part by receptor heteromerization. In a fibroblast-like cell line, AT1R blockade inhibits downstream signaling of β-AR, and vice versa, β-AR blockade inhibits signaling of AT1R (Barki-Harrington et al., 2003). Accordingly, heteromerization between AT1R and α2C-adrenergic receptor (α2C-AR) was proposed to trigger an atypical Gs-cAMP–PKA signaling and norepinephrine hypersecretion in transfected HEK293T cells (Bellot et al., 2015). AT1R-β2AR heterodimerization results in an enhancement of β-arrestin coupling to β2AR (Toth et al., 2017). Adrenaline alpha 1D–adrenergic receptor (α1D-AR) and AT1Rs can form heterodimers. This formation is greater in preeclamptic rats compared to control group, suggesting that this heteromer may play a role in preeclampsia (Gonzalez-Hernandez Mde et al., 2010).

Regarding receptors that belong to the depressor arm of the RAS, Leonhardt et al. (2017) described heterodimerization between AT2R and MasR, which mediates the Ang-(1-7)– or the AT2R agonist-induced CX3C chemokine receptor-1 messenger RNA expression in cultured astrocytes from mice (Leonhardt et al., 2017). AT2R-MasR interaction was also shown in obese Zucker rat kidney, and this heteromer promotes diuretic and natriuretic responses and NO generation (Patel et al., 2017). MasR-B2R heteromerization is constitutively present in Wistar rat mesenteric vascular beds and in human glomerular endothelial cells (Cerrato et al., 2016). Heteromerization between the Ang-(1-7) MasR and the bradykinin B2R results in a delayed sequestration of the MasR from the plasma membrane and an increase in the affinity ligand binding properties of MasR in HEK293T-transfected cells (Cerrato et al., 2016). Altogether, these changes in receptor functional characteristics may lead to long-lasting protective biological properties.

Alamandine, a new component of the RAS exerts protective effects similar to those displayed by Ang-(1-7) through the stimulation of the MrgDR (Lautner et al., 2013; Santos et al., 2019). MrgDR has been shown to heteromerize with the Mas-related GPCR member E receptor (MrgER) in HEK293T cells expressing MrgDR and MrgER, which induces a lower internalization of MrgDR compared to cells expressing MrgD alone and an increase in extracellular signal–regulated kinase 1/2 (ERK1/2) phosphorylation (Milasta et al., 2006).

Not only receptor heteromerization induces changes in receptor functionality. AT1R was the first receptor of the RAS to show to form homodimers (Hansen et al., 2004). An increase in AT1R homodimerization has been reported in monocytes from patients with hypertension, which correlated with an increased Ang II–dependent monocyte activation and adhesiveness (AbdAlla et al., 2004). Furthermore, this homodimerization is covalently crosslinked by factor XIIIA transglutaminase, an enzyme involved in stabilizing fibrin polymer. In fact, inhibition of this enzyme causes a reduction in AT1R homodimers (AbdAlla et al., 2004). AT2R and MasR have also been shown to form homodimers (Leonhardt et al., 2017).

Receptor homo-oligomerization has been shown to be present in different pathological situations. For instance, amyloid β induces AT2R oligomerization with the consequent Gαq/11 protein sequestration and dysfunction in a model of Alzheimer’s disease, thus contributing to the neurodegenerative process during the progression of Alzheimer’s disease (AbdAlla et al., 2009).

**Figure 1:**
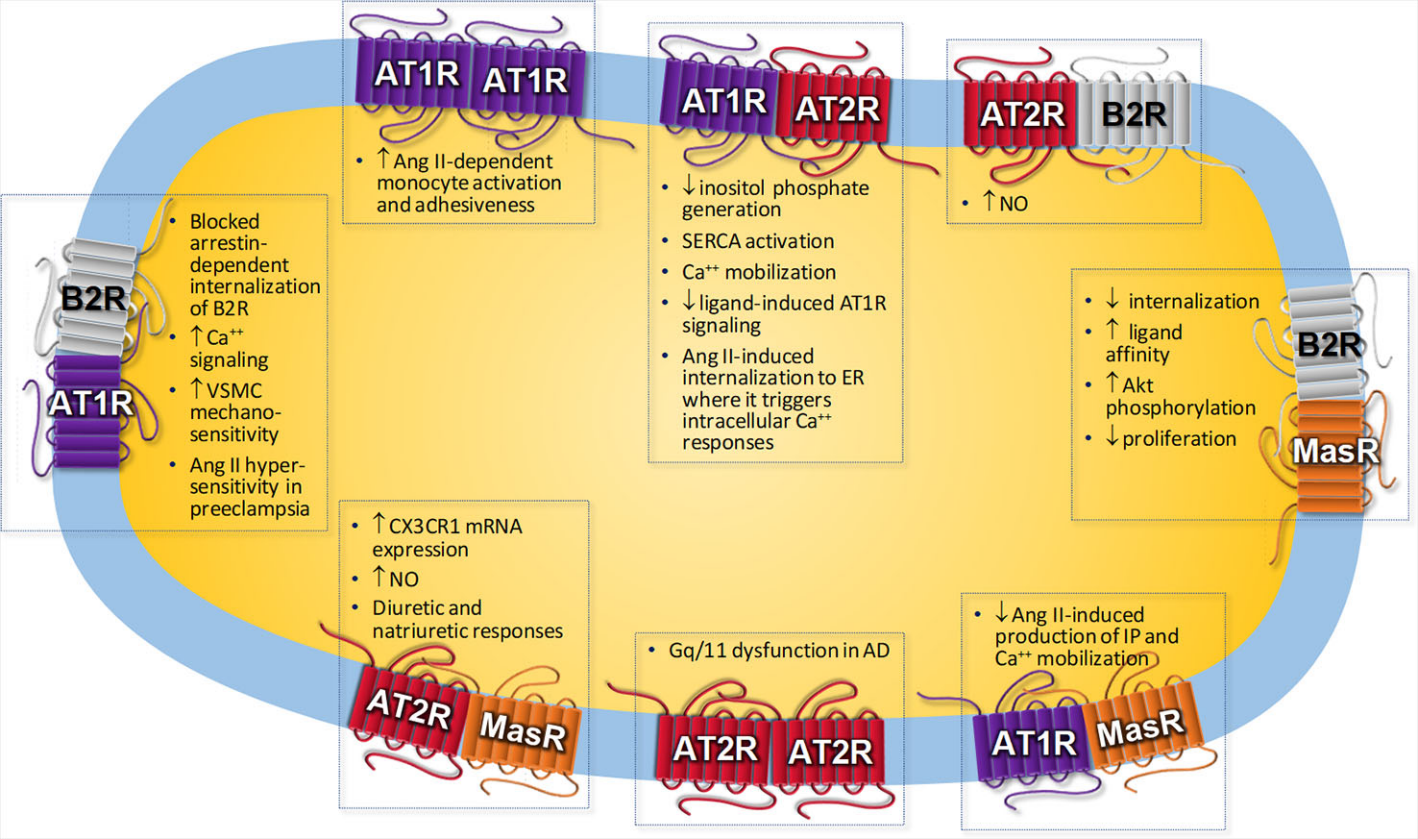
Biological responses associated to angiotensin receptors homo- or heteromerization. AD, Alzheimer’s disease; Ang II, Angiotensin II; AT1R, Ang II receptor type 1; AT2R, Ang II receptor type 2; B2R, Bradykinin receptor type 2; CX3CR1, CX3C chemokine receptor; ER, endoplasmic reticulum; IP, inositol phosphate; NO, nitric oxide; SERCA, sarco/endoplasmic reticulum Ca2+-ATPase; VSMC, vascular smooth muscle cells.

**Table 1** resumes Angs receptor heteromerization and its functional consequences. Some of the biological responses associated to AT1R, AT2R, and MasR heteromerization are represented in **Figure 1**. Given its fundamental role in physiological and pathological processes, GPCR oligomerization constitutes an important target in the development of novel drugs that would act through this class of receptors and could lead to a better design of new ligands, potentially more selective for these receptors and with greater binding capacity, with major implications in drug development and therapeutic approach in several diseases, such as cardiovascular disease.

**Table 1:** Angiotensin Receptors heteromerization and its functional consequences.

**Receptors**			**Model**	**Effect**	**Reference**
**AT1R**	–	AT1R	Monocytes from patients with hypertension	*Increased Ang II-dependent monocyte activation and adhesiveness*	AbdAlla et al., 2004
**AT1R**	–	APJR	Transfected CHO-K1 cells	*Diminished Ang II binding to AT1R*	Siddiquee et al., 2013
**AT1R**	–	AT2R	Rat fetal fibroblast; human myometrial biopsies	*Inhibition of inositol phosphate generation*	AbdAlla et al., 2001
**AT1R**	–	B2R	Transfected HEK293T cells	*Ang II hypersensitivity*	AbdAlla et al., 2000
**AT1R**	–	B2R	Primary aortic VSMC	*Blocked arrestin-dependent internalization of B2R*	Wilson et al., 2013
**AT1R**	–	B2R	Human placental biopsies	*Increased calcium signaling and high vascular smooth muscle mechanosensitivity*	Quitterer et al., 2019
**AT1R**	–	CB1R	Hepatic stellate cells from rats	*Enhanced calcium and mitogenic response to Ang II*	Rozenfeld et al., 2011
**AT1R**	–	CCR2	Transfected HEK293T and subtotal-nephrectomized rat (chronic kidney disease model)	*Enhanced β-arrestin2 recruitment* *Renal injury*	Ayoub et al., 2015
**AT1R**	–	D1R	Renal proximal tubule cells of WKY and SHR, A10 aortic vascular smooth muscle cells	*Decreased AT1R induced by D1R stimulation in WKY and SHR and A10 vascular smooth muscle cells* *Increased D1R induced by D1R stimulation only in WKY*. *D1R-AT1R interaction greater in WKY than in SHR* *D1R-AT1R interaction increased in WKY and decreased in SHR after D1R stimulation*	Zeng et al., 2003b
**AT1R**	–	D2R	Ethanol-administered rats	*Attenuated calcium and mitogenic response to Ang II*	Martinez-Pinilla et al., 2015
**AT1R**	–	D3R	Renal proximal tubule cells of SHR and WKY	*Decreased D3R induced by Ang II in both strains, being greater in SHR than in WKY* *Decreased AT1R in WKY by Ang II* *Increased AT1R in SHR by Ang II*	Zeng et al., 2003a
**AT1R**	–	D5R	Renal proximal tubule cells of SHR and WKY	*Negatively regulation of the expression of each receptor*. *Decreased D5R expression induced by Ang II in WKY and SHR*.	Zeng et al., 2005b
**AT1R**	–	ETBR	Renal proximal tubule cells of SHR and WKY	*Increased ETBR expression induced by Ang II in WKY cells. No change in SHR cells*	Zeng et al., 2005a
**AT1R**	–	FP	Primary rat aortic VSMCs	*Increased binding to AT1R*	Goupil et al., 2015
**AT1R**	–	MasR	Transfected HEK293T and mesenteric microvessels from wild type and MasR-KO mice	*Decreased Ang II-induced production of inositol phosphates and mobilization of intracellular calcium*	Kostenis et al., 2005
**AT1R**	–	P2Y6R	VSMCs from mice	*P2Y6 promotes Ang II–induced hypertension*	Nishimura et al., 2016
**AT1R**	–	SecR	Transfected CHO, HEK293T and COS-1 cells	*Negative allosteric modulatory impact on secretin-stimulated cAMP responses at SecR; Positive allosteric modulatory impact on secretin-stimulated cAMP responses at AT1R*	Harikumar et al., 2016
**AT1R**	–	α1DR	Aortic tissue from healthy and preeclamptic pregnant rats	*Higher heterodimerization in preeclamptic rats compared to control group*	Gonzalez-Hernandez Mde et al., 2010
**AT1R**	–	α2C-AR	Transfected HEK293T cells and mouse superior cervical ganglion neurons	*Atypical Gs-PKA signaling and norepinephrine hypersecretion*	Bellot et al., 2015
**AT1R**	–	β2AR	Transfected COS-7 and HEK293T cells	*Enhancement of β-arrestin coupling to β2AR*	Toth et al., 2017
**AT1R**	–	βAR	Mouse cardiomyocytes; HUVECs; transfected COS-7 and HEK293T cells	*Crossinhibition of signaling coupled to each receptor*	Barki-Harrington et al., 2003
**AT2R**	–	AT2R	Mice model of Alzheimer disease	*Gαq/11 sequestration and dysfunction*	AbdAlla et al., 2009
**AT2R**	–	B2R	Transfected PC-12 cells	*Enhancement of NO generation*	Abadir et al., 2006
**AT2R**	–	MasR	Cultured astrocytes from mice	*Induced CX3CR1 mRNA expression*	Leonhardt et al., 2017
**AT2R**	–	MasR	Obese Zucker rat kidney	*Increased NO generation* *Diuretic and natriuretic responses*	Patel et al., 2017
**MasR**	–	B2R	Transfected HEK293T cells	*Delayed sequestration of MasR from the plasma membrane* *Increased affinity ligand binding of MasR*	Cerrato et al., 2016
**MrgDR**	–	MrgER	Transfected HEK293T cells	*Decreased MrgDR internalization* *Increased ERK1/2 phosphorylation*	Milasta et al., 2006

Ang II, Angiotensin II; APJR, apelin receptor; AT1R, Ang II receptor type 1; AT2R, Ang II receptor type 2; B2R, Bradykinin receptor type 2; cAMP, Cyclic adenosine monophosphate; CHO, Chinese hamster ovary; CX3CR1, CX3C chemokine receptor; D1R, dopamine receptor type 1; D2R, dopamine receptor type 2; D3R, dopamine receptor type 3; D4R, dopamine receptor type 4; D5R, dopamine receptor type 5; ETBR, Endothelin receptor type B; FPR, Prostaglandin F receptor; HEK293T, human embryonic kidney 293T; HUVECs, Human umbilical vein endothelial cells; KO, knockout; MrgDR, Mas-related G protein-coupled receptor member D receptor; MrgER, Mas-related G protein-coupled receptor member E receptor; NO, nitric oxide; P2Y6R, P2Y6 purinergic receptor; PKA, protein kinase A; SecR, secretin receptor; SERCA, sarco/endoplasmic reticulum Ca2+-ATPase; SHR, Spontaneously hypertensive rat; VSMC, Vascular smooth muscle cells; WKY, Wistar Kyoto; α1DR, adrenergic α1D receptor; α2C receptor; α2C-AR, adrenergic; β2AR, adrenergic β2 receptor; βAR, adrenergic β receptor.

### Receptor Trafficking

To avoid the potential harms of prolonged agonist stimulation in the cell, GPCRs undergo a rapid internalization known as desensitization. Upon prolonged and/or repetitive agonist stimulation, GPCRs are internalized and trafficked inside the cell: They may be targeted to different organelles such as endoplasmic reticulum, Golgi body, mitochondria, or nucleus or recycled back to the plasma membrane or be degraded in lysosomes (Ribeiro-Oliveira et al., 2019).

GPCR desensitization is mediated by GRKs that phosphorylate the receptor, followed by recruitment of β-arrestins. GRKs mediate homologous desensitization, while other kinases such as PKA and PKC participate in the process of heterologous desensitization (Foster and Brauner-Osborne, 2018; Rajagopal and Shenoy, 2018). β-arrestins mediate both rapid uncoupling of G-protein and GPCR interaction and fast receptor internalization mediated by clathrin-coated pits (CCPs) (Sposini and Hanyaloglu, 2018).

Upon agonist stimulation, AT1R is internalized into early endosomes by a mechanism that requires the participation of CCP and caveolae and then recycled back to the cell surface in a process mediated by Rab4 and Rab11 porters in the early and late stages of the recycling process, respectively (Gaborik et al., 2001; Hunyady et al., 2002; Maxfield and McGraw, 2004; Szakadati et al., 2015; Bian et al., 2018). AT1R targeting to lysosome for degradation occurs under Rab7 overexpression (Dale et al., 2004). A decrease in AT1R endocytosis, which may result from a diminished receptor phosphorylation, enhanced activity and up-regulation of Rab 4 and Rab 11, or abnormal formation of endocytic vesicles, may be related to cardiovascular diseases development (Bian et al., 2018). In this sense, it has been proved that a GRK4 variant associated to essential hypertension decreases AT1R phosphorylation, thereby decreasing AT1R internalization (Chen et al., 2014). AT1R may be also degraded by the proteasome. Increased AT1R degradation has been documented after stimulation of the dopamine type 5 receptor (D5R) both in transfected HEK cells and in human renal proximal tubule cells. The mechanism involved dissociation of AT1R-D5R interaction and increase of glycosylated AT1R degradation *via* ubiquitin-proteasome system (Li et al., 2008).

**Figure 2:**
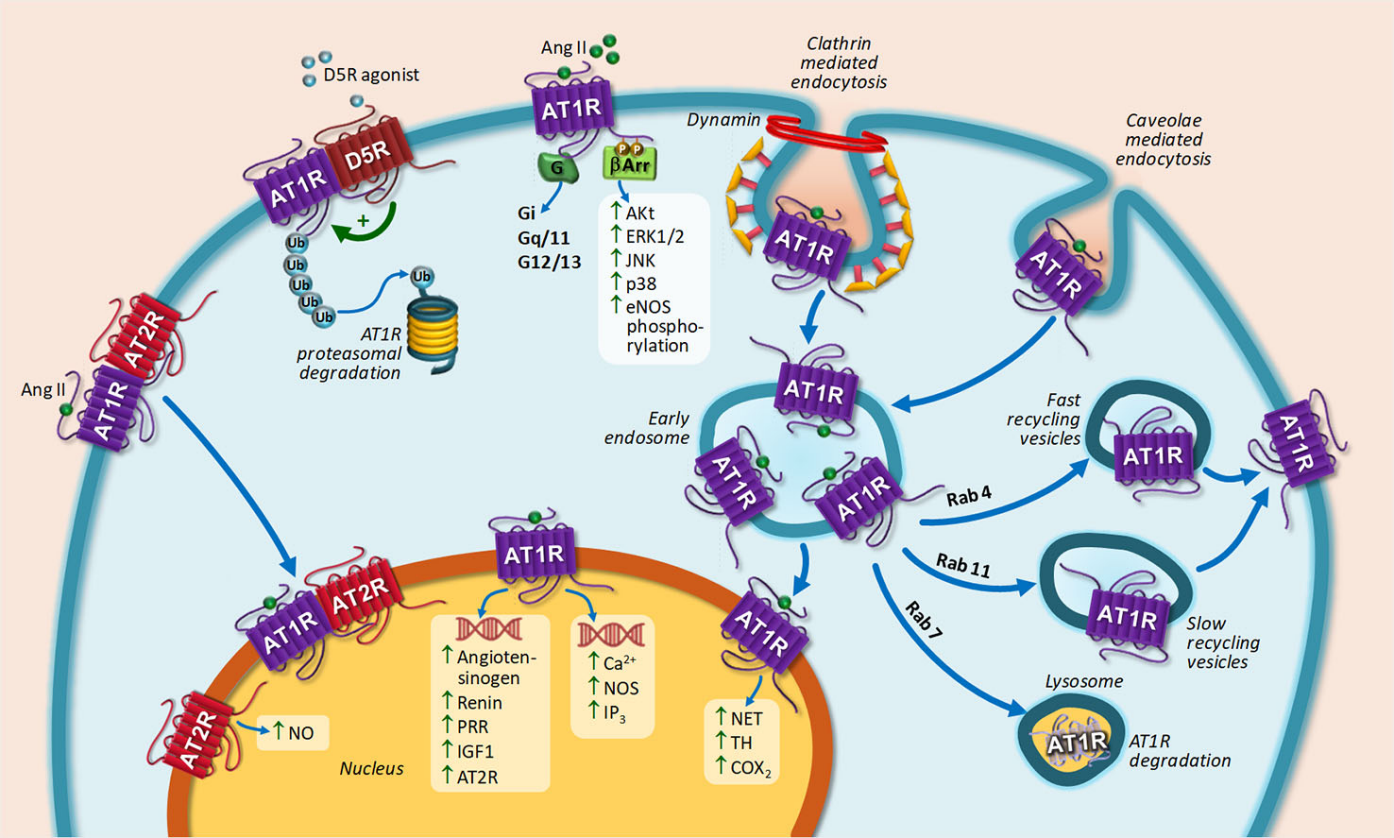
Intracellular trafficking of AT1R and main biological responses coupled to nuclear AT1R or AT2R stimulation. AT1R stimulation by Ang II induces G-protein activation, including Gi, Gq/11, and G12/13 (canonical signaling pathway) and G-protein-independent signal transduction (non-canonical pathway) leading to ERK 1/2, JNK, Akt, p38 mitogen-activated protein kinases activation, and eNOS phosphorylation through β-arrestin. Upon agonist stimulation, AT1R is internalized through CCPs and caveolae dependent pathways and then recycled back to the cell surface or targeted lysosome. AT1R-D5R heteromerization induced AT1R proteasomal degradation after D5R stimulation. AT1R translocation to the nucleus induces biological responses depicted in the scheme. AT2R translocation to the nucleus occurs by heteromerization with AT1R. Nuclear AT2R stimulation induced NO generation. Abbreviations: Ang II, Angiotensin II; AT1R, Ang II receptor type 1; AT2R, Ang II receptor type 2; D5R, dopamine receptor type 5; eNOS, endothelial nitric oxide synthase; ERK1/2, Extracellular signal-regulated kinase 1/2; IGF-1, insulin-like growth factor 1; IP3, Inositol triphosphate; JNK, Jun N-terminal kinase; NET, norepinephrine transporter; NO, nitric oxide; PRR, pro-renin receptor; TH, tyrosine hydroxylase; β-arr, β-arrestin.

Despite the fact that most GPCRs are internalized upon agonist stimulation, this is not the case for AT2R (Hein et al., 1997). It has been shown that AT2R are not internalized upon agonist stimulation in neuronal cells obtained from Wistar Kyoto rats (Lu et al., 1998). Instead, AT2R is internalized when a heteromer with AT1R is formed (Inuzuka et al., 2016). Upon Ang II stimulation, spatial arrangement of the complex is modulated in such a way that internalization of AT1R and AT2R occurs. AT2R is only internalized in the presence of AT1R. Losartan, an AT1R-specific antagonist, fully blocked both AT2R internalization together with AT1R (Inuzuka et al., 2016). **Figure 2** represents AT1R and AT2R trafficking upon agonist stimulation.

**Figure 3:**
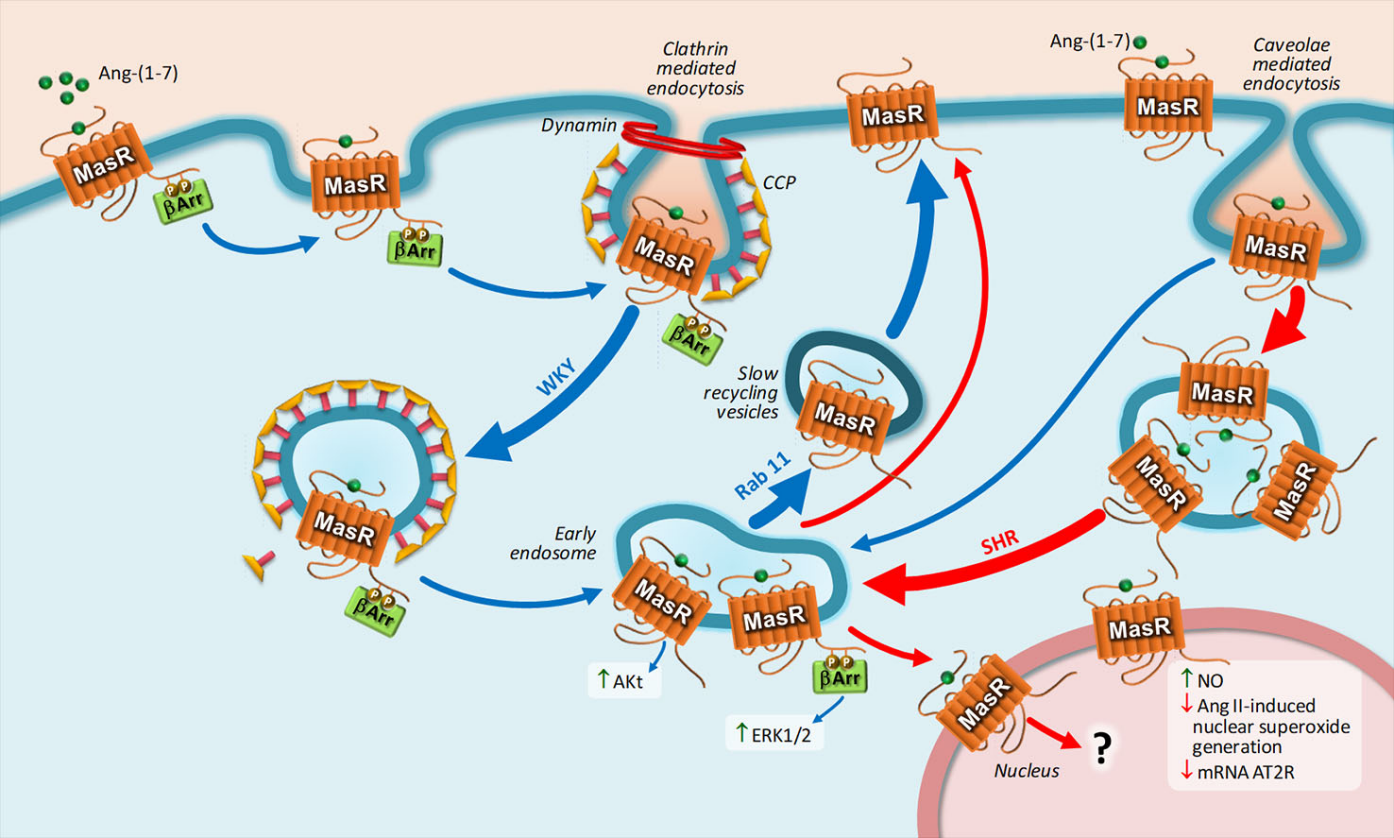
Intracellular trafficking of MasR and main biological responses coupled to nuclear MasR stimulation. Upon Ang-(1-7) stimulation, MasR is endocyted into early endosome by CCPs and caveolae in a dynamin-dependent pathway and then recycled back to the cell surface by slow recycling vesicles. Once in early endosome, MasR triggers Akt and ERK1/2 activation. MasR undergoes a unique trafficking in brainstem neurons from SHR: the number of MasRs internalized through caveolae was greater compared to that internalized by CCPs and the number of receptors that were returned to the cell membrane was smaller, resulting in a lower amount of resensitized MasRs present at the plasma membrane. A fraction of MasRs is translocated to the nucleus bound to its ligand only in brainstem neurons obtained from SHRs and not from normotensive rats. Upon stimulation, constitutive nuclear MasR stimulates NO generation and opposes the increase in nuclear superoxide production and the decrease in AT2R mRNA expression induced by Ang II. Blue and red arrows represent MasR trafficking in brainstem neurons Wistar-Kyoto (WKY) and spontaneously hypertensive rats (SHR), respectively. Abbreviations: Ang-(1-7), Angiotensin-(1-7); Ang II, Angiotensin II; AT2R, Ang II receptor type 2; ERK1/2, extracellular signal-regulated kinase 1/2; NO, nitric oxide; SHR, spontaneously hypertensive rat; WKY, Wistar Kyoto; β-arr, β-arrestin.

Regarding MasR, upon Ang-(1-7) stimulation, MasR is endocyted by CCP and caveolae in a mechanism dependent on dynamin, and then the receptor is directed to the cell surface by slow recycling vesicles (Cerniello et al., 2017). This mechanism of internalization was observed in transfected HEK293T cells and in cultured brainstem neurons from Sprague-Dawley rats, Wistar-Kyoto rats, and spontaneously hypertensive rats (SHRs) (Cerniello et al., 2017; Cerniello et al., 2019). The interesting observation is that MasR undergoes a unique trafficking in brainstem neurons from SHR: The number of MasRs internalized through caveolae was greater compared to that internalized by CCP, and the number of receptors that were returned to the cell membrane was smaller, resulting in a lower amount of resensitized MasRs present at the plasma membrane. Furthermore, a fraction of MasRs is translocated to the nucleus bound to its ligand only in brainstem neurons obtained from SHRs and not from normotensive rats (Cerniello et al., 2019). **Figure 3** represents MasR trafficking in brainstem neurons from both strains.

GPCR trafficking plays a fundamental role not only in signaling termination but also in regulating location and timing of receptor-mediated signaling process, altering in this way the ultimate cellular response (Lobingier and von Zastrow, 2019). In this sense, although endocytic trafficking has been classically considered as a process directed to mediate signal termination from the cell surface, studies over the past decade have brought up evidence about the existence of signaling platforms located at endosomes, establishing an intricate system to organize and regulate GPCR signaling (Hanyaloglu, 2018).

#### G Protein-Coupled Receptor Kinases

GRKs family is comprised by seven serine/threonine kinases that phosphorylate GPCRs upon binding of agonists to terminate GPCR-mediated signaling. GPCR phosphorylation changes the receptor conformation, exposing β-arrestin–binding domains of high affinity, which inhibit GPCR downstream signaling (Sun et al., 2018; Yu et al., 2018).

The GRK family has been classified into three subfamilies according to similarities in their structure and function: The subfamily of rhodopsin kinase (GRK1 and GRK7), the subfamily of β-adrenergic receptor kinase (GRK2 and GRK3), and the subfamily of GRK4 (GRK4, GRK5, and GRK6) (Metaye et al., 2005). GRK1 and GRK7 are expressed mainly in the retina, while GRK4 is predominantly expressed in the brain, kidney, testis, and human granulosa cells. GRK2, GRK3, GRK5, and GRK6 exhibit ubiquitous expression in the heart, brain, lung, kidney, and other tissues, where they regulate the functions of a variety of GPCRs.

Several lines of evidence indicate that GRKs are key modulators in GPCRs signaling. Given the high number of members of the GPCR family, each GRK is able to interact with and phosphorylate multiple receptors. GRKs also play a significant role in non-GPCR–mediated signaling, phosphorylating receptors that do not belong to the GPCR family, interacting with many cellular components or mediating cellular responses in a manner that not depends on phosphorylation (Sun et al., 2018; Yu et al., 2018).

All of these factors explain GRKs participation in a wide range of mechanisms of physiology and pathology (Sun et al., 2018). The development of mice lacking/overexpressing a defined GRK form constitutes the most useful technique to study the functions that individual GRKs play in an intact animal (Metaye et al., 2005). In particular, mice models of genetic modification of several members of the GRK family have shown their important function in cardiac physiology and pathology (**Table 2**). Additionally, several studies have supported the fundamental role of GRKs in animal models of heart diseases, such as hypertension and heart failure (HF) (**Table 3**) (Metaye et al., 2005; Traynham et al., 2016; Mayor et al., 2018).

**Table 2:** Animal models of GRKs genetic modifications affecting cardiac functions.

GRK studied	Animal model	Main findings	Reference
**GRK2**	GRK2 -/- KO mice	*Embryonic lethality*	Matkovich et al., 2006
	GRK2+/- mice	*Enhanced expression of key genes involved in physiological hypertrophy/cardioprotection* *Enhanced cardiac insulin sensitivity*	Lucas et al., 2014
	GRK2 cardiac overexpression	*Decreased β-adrenergic (β-AR) signaling (Attenuation of contractility and heart rate in response to a β-agonist)* *Desensitized AT1 receptor (AT1R)-mediated responses to angiotensin II (Ang II)*	Koch et al., 1995
	Transgenic mice with GRK2 vascular smooth muscle (VSM) targeted overexpression	*Increased blood pressure levels* *Cardiac hypertrophy*	Eckhart et al., 2002
**GRK4**	Mice carrying the naturally occurring polymorphism A142V in GRK4γ (polymorphism linked to hypertension in genetic studies)	*Development of hypertension* *Impaired diuretic and natriuretic effects of dopamine D1 Receptor agonists*	Felder et al., 2002
**GRK5**	Mice bearing targeted deletion of the GRK5 gene between exons 7 and 8 (GRK5-KO)	*Muscarinic supersensitivity and impaired receptor desensitization*	Gainetdinov et al., 1999
	Cardiac GRK5 overexpression	*Decreased β-AR signaling (Attenuation of contractility and heart rate in response to a β-agonist)* *No effects on AT1R signaling in response to Ang II*	Rockman et al., 1996

GRK, G protein-coupled receptor kinase.

**Table 3:** GRK alterations in animal models of cardiovascular disease.

Cardiovascular disease	Animal model	Main finding	References
**Heart failure (HF)**	Rats with surgical coronary artery occlusion	*Increased myocardial GRK2 and GRK5 mRNA expression and protein levels*	Vinge et al., 2001
	Spontaneously hypertensive heart failure (SHHF) rats	*Increased GRK2 expression and activity*	Yi et al., 2005
	Cardiac GRK2-deleted mice (deletion after birth) with surgical coronary artery occlusion	*Prevented maladaptive post-infarction remodeling and preserved β-adrenergic (βAR) responsiveness prior to coronary artery ligation*	Raake et al., 2008
	Paroxetine-mediated inhibition of GRK2 after myocardial infarction in mice	*Improved cardiac function, left ventricule (LV) structure and reverse the re-expression of the maladaptive fetal gene program that characterizes HF*	Schumacher et al., 2015
**Acute cardiac injury**	GRK2 inhibition through a C-terminal peptide that competes with GRK2 binding to Gβγ (βARKct)	*Enhanced cardiac function* *Increased sensitivity to acute* *β-AR stimulation*	Raake et al., 2013
	GRK2 overexpressing transgenic mice subjected to ischemia/reperfusion (I/R) injury	*Increased injury (i.e., cardiac infarction size)*	Brinks et al., 2011
	Fibroblast GRK2 KO subjected to I/R injury	*Decreased infarct size and preserved cardiac function*	Woodall et al., 2016
**Hypertension**	SHR rats	*Increased vascular smooth muscle GRK-2 protein expression*	Gros et al., 2000
	Hypertensive Dahl salt-sensitive rats	*Increased vascular GRK-2 protein expression*	Gros et al., 2000

GRK, G-protein–coupled receptor kinase.

One explanation regarding GRKs involvement in cardiac diseases pathophysiology may be related to GRKs participation in signaling pathways of the β-adrenergic, renin-angiotensin, and dopaminergic systems, all implicated in cardiovascular homeostasis and disease progression (Kim et al., 2005; Gurevich et al., 2016; Rudomanova and Blaxall, 2017; Mayor et al., 2018). In particular, several components of the family of GRKs have the ability to interact with AT1R (Turu et al., 2019). AT1R phosphorylation by GRK2 or GRK3 mediates the endocytosis of the receptor after homologous desensitization, while phosphorylation mediated by GRK5 or GRK6 affects receptor signaling through ERK1/2 activation in AT1R-transfected HEK293 cells (Kim et al., 2005). Interestingly, Kim et al. (2005) have shown that inhibition of GRK5 or GRK6 expression suppresses the activation of ERK that depends on β-arrestin, while GRK2/3 downregulation induces an increase in ERK signaling elicited by AT1R activation in HEK293 cells with heterologous expression of the receptor (Kim et al., 2005).

Regarding RAS involvement in cardiovascular diseases pathophysiology, sustained Gq-mediated Ang II AT1R activation has been proved to induce maladaptive cardiac hypertrophy that can lead to HF progression over time. GRK2 mediates Ang II–mediated cardiac contraction by interacting with Gαq, known as the final common trigger of maladaptive cardiac hypertrophy in situations of pressure overload (Schumacher et al., 2015). In association with this concept, Schumacher et al. (2015) demonstrated the effects of a peptide disrupting Gαq/GRK2 association on the suppression of pathological cardiac hypertrophy in an animal model of HF. It has also been proved that the selective serotonin reuptake inhibitor paroxetine, used as an antidepressant drug, selectively binds and inhibits GRK2 activity. In this context, treatment of mice with paroxetine was associated to improvement of heart function post-myocardial infarction (MI), being this benefit greater than that obtained with β-blockers treatment (Schumacher et al., 2015). Increased GRK2 expression in different cell types contributes to dysfunction of the heart and progression of cardiovascular disease (**Table 4**).

**Table 4:** Molecular changes and main effects associated to GRK2 overexpression in different cell types associated to cardiovascular disease.

Cell type	Molecular changes induced by GRK2 overexpression/activation	Main effects	Reference
**Cardiomyocytes**	Reduction of the contractile response to βAR stimulation	*Alteration of contractility*	Koch et al., 1995
	Impairment of insulin signaling cascades by interfering with insulin-Gq/11 signaling to GLUT4 translocation or by phosphorylating IRS1	*Impaired cardiac insulin sensitivity*	Lucas et al., 2014
	Regulation of Ang II–mediated contraction by directly interacting with Gαq	*Maladaptive remodeling and cardiac hypertrophy*	Rockman et al., 1996
	Increased mitochondrial superoxide and altered substrate utilization for energy production	*Metabolic dysregulation*	Sato et al., 2015; AbdAlla et al., 2016
	Impairment of the cardioprotective eNOS pathway	*Myocytes injury* *Alteration of cardiac function*	Huang et al., 2013
**Endothelial cells**	Impaired Akt/eNOS activation and lower NO synthesis/release	*Endothelial dysfunction*	Taguchi et al., 2014
**Fibroblasts**	Increased expression of TNF-α	*Fibrosis*	Woodall et al., 2016
**VSMC**	Attenuation of AR signaling and MAPK activation	*Increased resting blood pressure* *Vascular thickening* *Cardiac hypertrophy*	Eckhart et al., 2002

AR, adrenergic receptor; βAR, β-adrenergic receptor; eNOS, endothelial nitric oxide synthase; GLUT, glucose transporter; GRK, G protein-coupled receptor kinase; IRS, insulin receptor substrate; MAPK, Mitogen-activated protein kinase; NO, nitric oxide, TNF-α, tumor necrosis factor alpha; VSMC, vascular smooth muscle cells.

Altogether, the evidence indicates that modulation of GRK activity may have great potentiality in the design of new approaches for the treatment of pathologies related to GPCR dysregulation, such as cardiovascular disease (Mayor et al., 2018). Taking into account the evidence supporting the role of GRK2 activation in the cardiovascular pathology, the development of GRK2 inhibitors would be of particular interest for their potential use in clinical practice.

#### Arrestins

β-arrestins are scaffolding and multifunctional proteins that modulate GPCR functions and signaling (Sharma and Parameswaran, 2015). They are part of the arrestin family, which is classified into subfamilies on the basis of sequence homology and tissue distribution: visual rod arrestin and cone arrestin expressed in the eye and β-arrestins (β-arrestin1 and β-arrestin2) ubiquitously expressed (Peterson and Luttrell, 2017). β-arrestins are not only involved in G-protein desensitization but also in β-arrestin–dependent cell signaling in modulating GPCRs trafficking and in mediating transactivation and transcriptional regulation of the receptors (Bond et al., 2019). For further reading about the roles of arrestin, please refer to Gaborik et al. (2001); Peterson and Luttrell (2017), and Lymperopoulos (2018). β-arrestins participate in signal translation as scaffolds for transducer proteins that can trigger signals such as extracellular c-Jun N-terminal kinase (JNK), ERK 1/2, and MAPK, among others (Toth et al., 2018a). Related to this, the concept of biased agonism refers to β-arrestins capacity to initiate and regulate cellular signaling, implying that several different agonists for one GPCR can activate distinct subsets of downstream signaling pathways (Wang et al., 2018).

Based on the pleiotropic influence exerted on GPCRs, β-arrestins regulate a wide range of physiological functions, including apoptosis, organization of the cytoskeleton, polarity, and migration of cells, among others. In addition to their participation in a broad range of physiological processes, β-arrestins participate in the pathophysiology of numerous diseases, including inflammatory, cardiometabolic, and neurodegenerative diseases (Bond et al., 2019). Interestingly, both β-arrestin1 and β-arrestin2 display comparable biological functions in certain pathologies while they present opposite roles in other disorders (Sharma and Parameswaran, 2015).

AT1Rs play a fundamental role in heart diseases. AT1R stimulation by Ang II induces the activation of G-proteins, including Gi, Gq/11, and G12/13 (Forrester et al., 2018; Wang et al., 2018). However, Ang II also induces G-protein–independent signal transduction cascades, the so-called non-canonical pathways. Ang II induces Src tyrosine kinases, ERK1/2, JNK, Akt, and p38 MAPKs activation through β-arrestin2 (Forrester et al., 2018; Wang et al., 2018). Thus, in this way, both AT1R-mediated canonical and non-canonical pathways are involved in HF (Bond et al., 2019). It has been shown that the detrimental effects associated to AT1R activation are dependent on the Gq-protein pathway stimulation, while the beneficial effects are related to β-arrestin signaling activation (Kim et al., 2012; Atef and Anand-Srivastava, 2016). This evidence has been the basis for the development of biased agonists as new therapeutic agents in the treatment of HF (Boerrigter et al., 2012).

Biased agonism is a term used to make reference to the capacity of ligands that act on the same GPCR to evoke different cellular signaling pathways by preferentially inducing the stabilization of differential active states of receptor conformation (Wootten et al., 2018). In this context, TRV120027 (Sar-Arg-Val-Tyr-Ile-His-Pro-D-Ala-OH) is a β-arrestin biased AT1R ligand that has been shown to promote β-arrestin2 recruitment to AT1R in HEK293 cells overexpressing β-arrestin2 and AT1R, activating in this way the p42/44 MAPK and Src pathways and the phosphorylation of the endothelial NO synthase (Violin et al., 2010). Instead, TRV120027 antagonizes G-protein coupling to AT1R, which results in prevention of the increase in arterial pressure exerted by Ang II in rats, in the same way as losartan and telmisartan, unbiased AT1R antagonists (Violin et al., 2010). However, TRV120027 improved cardiac performance and conserved stroke volume, opposing the unbiased antagonists effects of decreasing cardiac performance (Violin et al., 2010).

AT1R-induced cardiac hypertrophy might be dependent on the activation of G-protein but not of β-arrestin, since β-arrestin biased agonist [Sar1,Ile4,Ile8]-Ang II (SII-Ang II) stimulation of AT1Rs did not induce hypertrophy in neonatal ventricular cardiomyocytes (Smith et al., 2011). Another β-arrestin biased agonist (TRV120067) improved mice cardiac structure and function due to stimulation of ERK1/2- ribosomal S3 kinase signaling elicited by β-arrestin/AT1R activation in an animal model of familial dilated cardiomyopathy (Ryba et al., 2017).

Not only kinases but also ion channels are regulated through β-arrestin upon AT1R activation. TRV120027 stimulates acute catecholamine secretion through coupling with the transient receptor potential cation channel subfamily C 3 in a β-arrestin1–dependent mechanism (Liu et al., 2017). AT1R stimulation induces β-arrestin1 recruitment and the subsequent internalization of CaV1.2 channels (Hermosilla et al., 2017) or β-arrestin2 recruitment followed by activation of L-type Ca2+ channels (Kashihara et al., 2017).

Ang-(1-7) also acts as a biased AT1R agonist. Ang-(1-7) counteracts the Ang II/AT1R/Gq pathway but stimulates β-arrestin recruitment to the AT1R, justifying, in part, its cardioprotective effects. Ang-(1-7) was shown to reverse phenylephrine-induced aorta contraction, an effect lost in KO-AT1R mice (Galandrin et al., 2016). Altogether, it seems that in cardiovascular diseases treatment, the ideal ligand for AT1R would be that one acting as an antagonist of the canonical G-protein pathway but at the same time as agonist of the receptor conformation promoting the non-canonical pathway through β-arrestin signaling. However, activation of AT1R-mediated β-arrestin signaling in adrenocortical zona glomerulosa cells may have cardiac detrimental effects because of aldosterone cardiotoxic actions (Maning et al., 2017). Increased levels of plasma aldosterone promote maladaptative cardiac remodeling and hypertrophy, along with a pro-inflammatory and pro-oxidant state with collagen deposition and fibrosis in the failing heart (Zhao et al., 2006). Adrenal β-arrestin stimulates aldosterone synthesis from adrenocortical zona glomerulosa cells through an AT1R-dependent mechanism, inducing its “second wave” of signaling to up-regulate the steroidogenic acute regulatory protein *via* ERK1/2 activation and also facilitating aldosterone secretion from adrenocortical zona glomerulosa cells (Lymperopoulos et al., 2011). Since Ang II–induced aldosterone production from the adrenal cortex is dependent on Gq-protein and β-arrestin1 activation, it has been proposed that complete inhibition of both signaling cascades is needed to fully block the synthesis of adrenal aldosterone in pathologies associated to high circulating levels of this hormone, such as HF (Maning et al., 2017). In this sense, AT1R blockers efficacy to block β-arrestin1 activation has been evaluated. Losartan has been demonstrated to be a poor biased antagonist, since it elicits a weak antagonism for β-arrestin1 activation by AT1R, being unable to prevent post-MI hyperaldosteronism in an animal model of HF (Lymperopoulos et al., 2011; Lymperopoulos et al., 2014). On the contrary, candesartan and valsartan are the most potent β-arrestin1 inhibitors, with an excellent efficacy to decrease aldosterone levels *in vitro* and *in vivo*, being the chosen agents of their class to treat HF (Dabul et al., 2015).

β-arrestin signaling induced by AT1R activation in astrocytes might contribute to central control of blood pressure and may be implicated in the pathophysiology of hypertension, given the fact that AT1R signaling through β-arrestins may be involved in the regulation of angiotensinogen production by these cells (Negussie et al., 2019).

Given their central role in the regulation of GPCRs signaling, manipulating β-arrestin function may be a key factor in the development of novel strategies for the treatment of several diseases. β-arrestin activity impairment may enhance G-protein signaling in situations where β-arrestin blockade may be deleterious, such as in inflammatory and neurodegenerative diseases and cancer (Hu et al., 2013; Urs et al., 2015). On the opposite, selective activation of β-arrestin–dependent signaling may be beneficial in situations in which excessive GPCR stimulation underlies a pathophysiologic process, as is the case with AT1R activation by Ang II in cardiovascular disease (van Gastel et al., 2018). In this sense, β-arrestin biased ligands may be postulated as new therapeutic agents that could selectively activate some beneficial signaling pathways while avoiding the untoward activation of G-proteins, which has been shown to be detrimental and involved in cardiovascular disease development (Wang et al., 2018).

#### GPCR Endosomal Signaling

Evidence from several studies has proven that GPCRs in endosomes can continue signaling after internalization. This led to the hypothesis of the existence of a third trafficking pathway, apart from degradation or recycling, in which GPCR can remain on intracellular membranes such as endosomes for longer periods of time (Retamal et al., 2019). At this location, GPCR can elicit distinct β-arrestin and G-protein–dependent signaling processes. GPCR intracellular localization can strongly contribute to receptor signaling with important consequences in health and disease, opening up the possibility to design new drugs taking into account of the context of where the receptor is signaling from (Jong et al., 2018).

We have previously shown in MasR-transfected HEK293T cells that upon agonist stimulation MasR is internalized into early endosomes. Once it has been internalized, MasR promote the activation of Akt in a mechanism that does not depend on β-arrestin2. Conversely, the activation of ERK1/2 depends on β-arrestin2. Afterwards, MasR returns back to the cell membrane through slow recycling vesicles (Cerniello et al., 2017) (**Figure 3**).

Regarding AT1R, β-arrestin binding to AT1R induces receptor endocytosis in transfected HEK293T cells (Toth et al., 2017; Toth et al., 2018b). After GRK-phosphorylation of AT1R, β-arrestin binds the complex formed between the phosphorylated AT1R and GRK, which is targeted to early endosomes. AT1R preferentially fuses to Rab5-endosomes, which favors its retention in early endosomes, preventing recycling and degradation and also prolonging the intracellular effects of Ang II. Sustained binding of AT1R to β-arrestin induces trafficking to late endosomes and lysosomes, promoting receptor down-regulation and terminating intracellular signaling pathways activated by Ang II/AT1R (Dale et al., 2004; Toth et al., 2018b).

## GPCRs in the Nucleus

Until recently, it was thought that GPCR signaling came exclusively from the plasma membrane in response to extracellular stimuli. The first evidence suggesting the existence of GPCRs in the nucleus comes from the 1980s, with the demonstration of AC localization and activity in the nuclear fraction of the cell, which represented around 30% of total cellular AC activity (Monneron and D’Alayer, 1980; Buchwalow et al., 1981). At that time, however, the existence of enzymatic activity in the nucleus could not be explained. Recent research has brought to light the existence of nuclear GPCRs with the capacity to initiate identical and/or different signaling pathways compared to their respective counterparts located on the cell surface (Ribeiro-Oliveira et al., 2019). Approximately 30 different types of GPCRs have been detected in the nuclear membrane of multiple cells (Gobeil et al., 2006; Zhu et al., 2006). The first receptors described to be present were muscarinic cholinergic receptors in the nucleus of keratocytes, epithelial and endothelial cells (Lind and Cavanagh, 1993), and prostaglandin E receptor in nuclei isolated from adult rat liver and newborn porcine brain cortex (Bhattacharya et al., 1998).

The nuclear membrane is a double lipid membrane: the inner and the outer nuclear membrane. The nuclear pore complex allows the selective exchange of macromolecules and RNA between the cytoplasm and the nucleoplasm (Isermann and Lammerding, 2013). Nuclear GPCRs can be located at the inner and/or outer nuclear membranes of the nuclear envelope with an orientation that allows the exposure of the effector-binding domain either to the cytosol or nucleus. It has also been postulated that nuclear GPCRs could be located in the nucleoplasm, particularly in the network of invaginations of the inner and outer nuclear membranes (Jong et al., 2018; Bhosle et al., 2019; Ribeiro-Oliveira et al., 2019). AT1R and MasR have been described to colocalize with a nuclear pore complex marker in brain neurons, suggesting the presence of both receptors in the nuclear pore complex (Lu et al., 1998; Cerniello et al., 2019). In cardiomyocytes, nuclei GPCRs have been described to be present in both inner and outer membranes (O’Malley et al., 2003; Jong et al., 2005; Vaniotis et al., 2011; Bkaily et al., 2012; Tadevosyan et al., 2012). Nuclear α1- and β1-ARs, endothelin, and Ang receptors have been shown to activate intranuclear signaling in isolated nuclei from cardiac myocytes, suggesting that these receptors would be located in the inner membrane (Tadevosyan et al., 2010; Wright et al., 2012; Vaniotis et al., 2013). In fact, direct evidence from confocal microscopy and immunocytochemistry has shown that nuclear α1-ARs from cardiac myocytes are localized in the inner membrane with such an orientation that allows intranuclear signaling activation (Wu et al., 2014). On the other hand, indirect evidence arising from the activation of different signaling pathways suggests that some nuclear GPCRs are located in the outer membrane. For instance, nuclear endothelin receptors activate phospholipase C-ϵ in the outer nuclear membrane from nuclei isolated from cardiac myocytes (Zhang et al., 2013).

The nucleus possesses all fundamental components involved in GPCRs signaling pathways such as G-proteins, second messengers, ion channels, and regulator molecules (Ribeiro-Oliveira et al., 2019). Nuclear GPCRs mainly regulate nuclear Ca^2+^, NO levels, or cAMP synthesis; however, others, such as IP3, cyclic guanosine monophosphate, and diacylglycerol have been described. The presence of AC, phospholipase A2, phospholipase C-β1, phospholipase D, and β-arrestin1 have also been found in the nucleus membrane (Booz et al., 1992; Kim et al., 1996; Yamamoto et al., 1998; Topham et al., 1998; Faenza et al., 2000; Zhang et al., 2001; Scott et al., 2002; Fatima et al., 2003; Wang et al., 2003; Boivin et al., 2006; Gayral et al., 2006; Vaniotis et al., 2013). Ca^2+^ATPase sensitive to ryanodine or inositol triphosphate have also been reported to be present in the nuclear membrane (Guihard et al., 1997; Abrenica and Gilchrist, 2000; Bare et al., 2005).

Second messengers modify diverse cellular processes and reactions, such as DNA transcription, cell proliferation, redox status, and genesis of tumors (Jong et al., 2018; Ribeiro-Oliveira et al., 2019). In addition, proteins that act as regulators of G-protein signaling can stay in the nucleus or commute between the nucleus and the cytosol or some are directed to specific subnuclear locations (Huang and Fisher, 2009; Huang and Fisher, 2009). It has been demonstrated that several nuclear receptors such as B2R, endothelin-1 receptor, and AT1R regulate Ca^2+^ concentration in the nucleoplasm, constituting important modulators of nucleoplasmic Ca^2+^ transients (Kockskämper et al., 2008; Savard et al., 2008; Tadevosyan et al., 2017).

GPCRs have been found in the nuclei of cardiomyocytes, such as endothelin B receptor, AT1R, AT2R, and ARs (Boivin et al., 2003; Huang et al., 2007; Tadevosyan et al., 2010; Vaniotis et al., 2011; Merlen et al., 2013). Endothelin B receptor is involved in the activation of inositol triphosphate (IP3) and nuclear Ca^2+^ mobilization (Merlen et al., 2013). Nuclear AT1R and AT2R mediate the novo synthesis of mRNA affecting nuclear factor κB gene transcription, while nuclear AT1R induces Ca^2+^ transients *via* IP3 receptor-dependent pathways (Tadevosyan et al., 2010). α1-AR induces PCK activation and regulates contractile function in adult cardiac myocytes (Wu et al., 2014). β1-AR receptor activates AC, probably through Gαs, while β3-AR stimulation activates NO generation, probably through Gαi, and gene transcription (Boivin et al., 2006; Vaniotis et al., 2013).

The presence of nuclear GPCRs has been suggested to occur in at least three different ways: (1) agonist dependent or independent translocation from the cell membrane, (2) it might be synthesized in the endoplasmic reticulum and then traffic directly to the nucleus by lateral diffusion, (3) or be synthesized within the nucleus (Bhosle et al., 2019; Ribeiro-Oliveira et al., 2019). The involvement of small GTPases, importins, and sorting nexin proteins has been postulated in the process of translocation of nuclear GPCRs mediated by vesicles (Bhosle et al., 2019). GPCR translocation to the nucleus depends for some receptors on a specific sequences of short peptides containing residues of basic amino acids (usually repetitions of lysine/glycine-arginine sequences) in the C terminus or an intracellular loop of the GPCR, the nuclear localization sequences (NLSs) (Bhosle et al., 2019; Ribeiro-Oliveira et al., 2019). AT1R translocation to the nucleus depends on the NLS sequence present in AT1R (Lu et al., 1998; Morinelli et al., 2007). In contrast, Ang II did not induce AT2Rs translocation to the nucleus, since they do not have the putative NLS sequence (Lu et al., 1998; Costa-Besada et al., 2018). Instead, upon Ang II stimulation, the promyelocytic zinc finger protein, which acts as a transcription factor, binds to AT2R C-terminal peptide, resulting in AT2R accumulation in the perinuclear region (Senbonmatsu et al., 2003). AT2R translocation to the nucleus occurs only by heteromerization with AT1R (Inuzuka et al., 2016).

The process of translocation of GPCRs to the nucleus seems to depend on the specific cell type and also on conditions of the cell, such as its metabolic state, interaction with other receptors, or even a pathological state (Ribeiro-Oliveira et al., 2019). It also depends on the type of receptor. For instance, Ang II induces nuclear sequestration of AT1R in rat brain neurons but not in rat astroglial cells and VSMC (Lu et al., 1998; Morinelli et al., 2007). Ang II–induced nuclear translocation of AT1R mediates neuromodulatory chronic effects of this peptide in hypothalamic and brainstem neurons (Lu et al., 1998) and activation of cyclooxygenase 2 gene transcription in HEK293 cells stable expressing wild type AT1R (Morinelli et al., 2007). MasR is another example of cell specific receptor nuclear translocation. Agonist stimulation induces MasR translocation to the nucleus in brainstem neurons from SHR but not from normotensive rats (Cerniello et al., 2019). Physiological consequences of agonist-dependent MasR trafficking to the nucleus only in neurons from SHR need to be elucidated.

Ang receptors can be constitutively located at the nuclear/perinuclear membranes, independent of agonist stimulation. Regarding these receptors, it has been shown that direct activation of nuclear AT1R results in an increase in the intranuclear free Ca^2+^ in the nucleus of human VSMC (Bkaily et al., 2003) and in reactive oxygen species generation in the nucleus of cells from rat renal cortex (Pendergrass et al., 2009). AT1R present in rat ventricular cardiomyocytes nuclear membranes couples to RNA transcription and nuclear calcium signaling, and, in this way, nuclear AT1R signaling constitutes a fundamental intermediary of Ang II effects in the promotion of cardiac remodeling through alteration of gene transcription (Tadevosyan et al., 2010). AT1R binding sites present in the nuclei of canine atrial fibroblast are coupled to the mobilization of intranuclear Ca^2+^, regulating, in this way, distinct processes such as cell proliferation, gene expression of collagen, and also its secretion (Tadevosyan et al., 2017). Ang II can activate nuclear AT1R in human mesangial cells by an intracrine mechanism that does not depend on plasma membrane Ang II receptors (da Silva Novaes et al., 2018). In rat renal cortical cells, Ang II binds to nuclear AT1Rs to induce the transcription of the transforming growth factor-β, macrophage chemoattractant protein-1, and the sodium and hydrogen exchanger-3 to regulate responses associated to tubular sodium transport, cellular growth, and inflammation (Li and Zhuo, 2008).

Having a look at the RAS depressor arm, it has been reported that nuclear AT2R and MasR are functionally linked to the production of NO (Gwathmey et al., 2009; Gwathmey et al., 2010; Tadevosyan et al., 2017). Ang-(1-7) stimulates NO generation through nuclear MasR stimulation in nuclei from kidneys of young adult sheep (Gwathmey et al., 2010) and substantia nigra of rodents (Costa-Besada et al., 2018), but these effects were independent of MasR translocation to the nucleus. The nuclear MasR-mediated increase in NO is reduced in old animals and also in fetally programmed hypertensive animals (Gwathmey et al., 2012; Chappell et al., 2014; Costa-Besada et al., 2018). Ang-(1-7)–induced nuclear activation of MasR not only exerted an elevation in NO generation in the nucleus of animal neurons but also opposes the increase in nuclear superoxide production and the decrease in AT2R mRNA expression induced by Ang II. This function is impaired in aged animals (Costa-Besada et al., 2018).

Nuclear GPCRs can be activated by endogenous ligands synthetized within the cell or can be activated in a constitutive way. Ligands from the extracellular space might reach those receptors by cellular uptake using selective membrane transporters, membrane exchangers, or *via* receptor endocytosis (Ribeiro-Oliveira et al., 2019). In fact, the components of the RAS are present in the nucleus of several cells types (Tang et al., 1992; Jimenez et al., 1994; Camargo de Andrade et al., 2006; Cook and Re, 2012; Gwathmey et al., 2012; Kumar et al., 2012; Alzayadneh and Chappell, 2015; Costa-Besada et al., 2018; Cerniello et al., 2019). Thus, nuclear Ang receptors may be activated by endogenously synthetized Angs (Tadevosyan et al., 2012). Ang II is generated intracellularly (Singh et al., 2007; Kumar et al., 2009). In this way, Ang II induces autocrine biological responses by interacting with cytoplasmic proteins or receptors present in the nucleus, thus regulating gene expression (Re et al., 1984; Tadevosyan et al., 2010; Cook and Re, 2012; Tadevosyan et al., 2017). Intracellular Ang II synthesis has been demonstrated in cardiomyocytes and fibroblasts (Singh et al., 2007; Singh et al., 2008). Both angiotensinogen and renin can be synthetized locally or taken up from circulation (Kumar et al., 2009). Angiotensin-converting enzyme (ACE) is also expressed in cardiomyocytes and can be localized within the cell including the cytoplasm, endoplasmic reticulum. and nucleus (Vidotti et al., 2004; Camargo de Andrade et al., 2006; Shen et al., 2008). Lucero et al. (2010) have shown that ACE transited through the early endosome, the late endosome, and the lysosome and was directed to VSMC and endothelial cells nuclei. These results revealed the pathway employed by these cells to deliver ACE coming from the extracellular space to the nucleus (Lucero et al., 2010).

Ang II generation has been demonstrated to occur in cardiomyocytes cytoplasm, involving renin and chymase. In this sense, intracellular Ang II generation was fully blocked by renin and chymase and not by ACE inhibitors, suggesting that ACE is not involved in Ang II synthesis within cardiomyocytes (Kumar et al., 2009). Intracellular Ang II disrupts cell proliferation and signal transduction and elevates blood pressure (Ellis et al., 2012). Ang II concentration inside the cell is up-regulated in certain diseases, including hypertension and diabetes (Frustaci et al., 2000; Serneri et al., 2001; Singh et al., 2007). Intracellular Ang II in myocytes may reflect disease severity, given the fact that the increase progresses along with the disease (Serneri et al., 2001). Ang II levels in cardiomyocytes are increased in diabetic rats, being 10-fold higher than those found in healthy rats (Singh et al., 2008). In cardiomyocytes, the increase in intracellular Ang II production induced by high glucose concentrations is mediated by chymase rather than ACE (Singh et al., 2008). Intracellular Ang II levels are increased more than three times in myocytes from human diabetic patients and two times in diabetic hypertensive patients in comparison to diabetic nonhypertensive patients (Frustaci et al., 2000). Intracellular Ang II may contribute to disease progression by enhancing oxidative damage, cardiac cell apoptosis, and necrosis. Moreover, mice overexpressing Ang II only in cardiomyocytes developed hypertrophy, suggesting that intracellular Ang II induces cardiac hypertrophy (Baker et al., 2004). Altogether, these results demonstrate that an intracellular RAS exists in cardiac cells. In addition, this intracellular RAS may act as an autocrine system, acting on receptors present in the nucleus.

Alterations in nuclear GPCRs density have been reported in pathophysiological conditions (Ribeiro-Oliveira et al., 2019). Regarding the RAS, it has been shown that atrial-fibroblast nuclear ATRs are altered in congestive HF: an increment in intracellular Ang II and nuclear AT1R expression, together with the alteration in nuclear AT2Rs glycosylation. The increased amount of AT1R in atrial fibroblasts was associated with alteration of the expression and secretion of collagen and with changes in cell proliferation (Tadevosyan et al., 2017). By contrast, in established hypertension, nuclear AT1R expression from rat renal cortex was lower compared to normotensive conditions, despite the fact that AT1R levels were predominant in the nuclear fraction vs. the plasma membrane (Pendergrass et al., 2006). Regarding MasR, we have shown that they are expressed in nuclear membranes from brainstem neurons of normotensive and SHR rats, but those levels were similar in boths strains (Cerniello et al., 2019).

**Table 5** resumes nuclear Ang receptors and their biological response. **Figures 2** and **3** represent main biological responses of AT1R, AT2R and MasR present in the nucleus.

**Table 5:** Nuclear angiotensin receptors.

Receptor	Biological response	Cell/tissue	Reference
**AT1R**	Chronic neuromodulatory actions of Ang II (**↑** NET transcription)	*Hypothalamic and* *brainstem primary rat neurons*	Lu et al., 1998
	**↑** nuclear Ca^2+^ levels	*Human vascular smooth* *muscle cells*	Bkaily et al., 2003
	Not determined	*HEK transfected cells*	Lee et al., 2004
	**↑** reactive oxygen species production	*Rat renal cortex nucleus*	Pendergrass et al., 2009
	Not determined	*Rat vascular smooth muscle cell line*	Cook et al., 2006
	**↑** nuclear calcium levels	*Fetal human endocardial endothelial cells*	Jacques et al., 2003
	Not determined	*Rat renal cortex from control and Ang II-infused rats*	Licea et al., 2002
	**↑** cyclooxygenase 2 transcription	*HEK transfected cells*	Morinelli et al., 2007
	**↑** nuclear Ca^2+^ levels	*Rat ventricular cardiomyocytes*	Tadevosyan et al., 2010
	**↑**TGF-beta1, MCP-1, and NHE-3 mRNAs	*Rat renal cortical cells*	Li and Zhuo, 2008
	Not determined	*Rat liver*	Re et al., 1984; Tang et al., 1992; Jimenez et al., 1994
	**↑** nuclear IP_3_ and Ca^2+^ levels **↑** collagen‐1A1 mRNA	*Canine atrial fibroblasts*	Tadevosyan et al., 2017
	Overexpression of fibronectin ↑ cell proliferation	*Human mesangial cells*	da Silva Novaes et al., 2018
	**↑** AT2R, angiotensinogen, renin, and prorenin/renin receptor mRNA **↑** NOX4/superoxide **↑** IP3/Ca^2+^ levels **↑** PGC-1α **↑** IGF-1	*Dopaminergic neurons*	Villar-Cheda et al., 2017
**AT2R**	Not determined	*Fetal human endocardial endothelial cells*	Jacques et al., 2003
	**↑** NO generation	*Dopaminergic neurons*	Villar-Cheda et al., 2017
**MasR**	Not determined	*Brainstem neuronal culture from normotensive and SHR rats*	Cerniello et al., 2019
	**↑** NO generation	*Kidneys of young adult sheep*	Gwathmey et al., 2010
	**↑** NO generation Counteraction of the increased Ang II-derived nuclear superoxide generation **↓** mRNA for AT2Rs	*Rat substantia nigra*	Costa-Besada et al., 2018

IGF-1, insulin-like growth factor 1; MCP-1, macrophage chemoattractant protein-1; NHE-3, sodium and hydrogen exchanger-3; NET, norepinephrine transporter; NO, nitric oxide; PGC-1α, peroxisome proliferator-activated receptor gamma coactivator 1-alpha; TGF-beta1, transforming growth factor-beta1.

## Conclusions

Receptor trafficking is a key event in the ultimate cellular response elicited by receptor stimulation. Upon agonist stimulation, the receptor activates different signaling pathways from the plasma membrane, but once internalized it may be recycled back to the cell membrane or be directed to different organelles leading to other signaling events or be directed to lysosomes for degradation. Thus, the biological response may result from the integration of those events. In addition, receptor functionality may also be influenced by interaction with other receptors, leading to different biological responses compared to the receptor alone. Receptors are in constant dynamism, undergoing dynamic interactions with each other and with G-proteins, as well as with the surrounding cytoskeleton, other structural components, and other receptors, leading to the formation of receptor heteromers. Altogether, this explains the diversity in receptor function. Broadening our knowledge on receptors regulation would open new therapeutic strategies. There is a need to go far beyond the concept of designing drugs to activate or inhibit a single GPCR to design newer drugs directed to regulate a specific receptor signaling pathway or effector at a desired time and subcellular location. Particularly, targeting of Ang receptors-dependent signaling constitutes one of the most promising tools in the therapy of cardiovascular disease and needs further investigation.

## Author Contributions

NR, MS, AP, and MG wrote the manuscript. MG coordinated and revised the manuscript.

## Funding

This work was supported by grants from Universidad de Buenos Aires [20020160100134BA] and Agencia Nacional de Promoción Científica y Tecnológica [2016-2978].

## Conflict of Interest

The authors declare that the research was conducted in the absence of any commercial or financial relationships that could be construed as a potential conflict of interest.

## Abbreviations

AC, Adenylate Cyclase; Ang, Angiotensin; AT1R, Angiotensin II type 1 receptor; AT2R, Angiotensin II type 2 receptor; B2R, Bradykinin type 2 receptor; α-AR, α-adrenergic receptor; β-AR, β-adrenergic receptor; βARK, β-adrenergic receptor kinase; cAMP, Cyclic adenosine monophosphate; CCPs, Clathrin-coated pits; D2R, Dopamine type 2 receptor; D5R, Dopamine type 5 receptor; ERK1/2, Extracellular signal-regulated kinase ½; GPCR, G-protein–coupled receptor; GRK, G-protein–coupled receptor kinase; HEK, Human embryonic kidney; HF, Heart failure; IP3, Inositol triphosphate; JNK, Jun N-terminal kinase; MasR, Receptor Mas; MAPK, Mitogen-activated protein kinase; MI, Myocardial Infarction; MrgDR, Mas-related G-protein–coupled receptor member D receptor; NLS, Nuclear localization sequences; NO, Nitric oxide; PKA, Protein kinase A; PCK, Protein kinase C; RAS, Renin-angiotensin system; SHR, Spontaneously hypertensive rats; VSMC, vascular smooth muscle cells.
